# Obese trauma patients are at increased risk of early hypovolemic shock: a retrospective cohort analysis of 1,084 severely injured patients

**DOI:** 10.1186/cc11334

**Published:** 2012-05-08

**Authors:** Jana Nelson, Adrian T Billeter, Burkhardt Seifert, Valentin Neuhaus, Otmar Trentz, Christoph K Hofer, Matthias Turina

**Affiliations:** 1 Department of Trauma Surgery, University of Zurich Hospital, Rämistrasse 100, CH-8091 Zurich, Switzerland; 2 Price Institute of Surgical Research, University of Louisville Hospital, 511 S Floyd Street, Louisville, KY 40201, USA; 3 Division of Biostatistics, Institute for Social and Preventive Medicine, University of Zurich, Hirschengraben 84, CH-8001 Zurich, Switzerland; 4 Department of Orthopaedic Surgery, Massachusetts General Hospital, 55 Fruit Street, Boston, MA 02114, USA; 5 Department of Anesthesiology, Stadtspital Triemli Zurich, Birmensdorferstraße 497, CH-8063 Zurich, Switzerland; 6 Department of Colorectal Surgery, Cleveland Clinic, 9500 Euclid Avenue, Cleveland, OH 44195, USA

## Abstract

**Introduction:**

Morbid obesity and its consequences are considered risk factors for adverse outcome in trauma, although the pathophysiologic mechanisms are incompletely understood. The aim of this study was to compare initial resuscitation, treatment, and short-term outcome of severely injured patients by body mass index (BMI).

**Methods:**

A total of 1,084 severely injured patients with an injury severity score of 16 or greater were enrolled between 1996 and 2009 and grouped according to BMI. Their course of treatment and in-hospital outcome were analyzed by univariate and multivariate comparison.

**Results:**

Of these patients, 603 (55.6%) were of normal weight with a BMI between 18.5 and 24.9, 361 (33.3%) had BMI values between 25 and 29.9, and 90 patients (8.3%) were obese (BMI ≥ 30). Thirty patients (2.8%) had BMI levels below 18.5. All groups were comparable with respect to injury severity, initial resuscitation, and time to ICU admission. There was a tendency towards higher mortality in obese patients (mortality 24.4%) and also overweight patients (mortality 18.8%) when compared with patients with a normal BMI (mortality 16.6%). Obese patients showed the highest mortality on day 0 (8.9% vs. 2.8% in the normal-weight group, *P *= 0.023), mostly due to persistent shock (6.7%). When corrected for BMI, obese patients are provided significantly lower volumes of intravenous fluids during the initial resuscitation period.

**Conclusion:**

In contrast to the mostly American literature, only a low percentage of trauma patients at a European trauma center are obese. These patients are at risk of higher mortality from persistent hemorrhagic shock in the initial phase after trauma, which may potentially be related to relative hypovolemia during the resuscitation period. In the later course of treatment, no significant differences exist with respect to specific complications, hospital stay, or in-hospital mortality.

## Introduction

Despite geographical differences, obesity has become a major burden to healthcare systems worldwide, especially in western countries. In North America, the prevalence of obesity (body mass index (BMI) ≥ 30), as defined by the World Health Organization [1], exceeded 20% in all but two states in 2009, and obesity is reported to now account for up to 9% of healthcare costs [2]. A similar trend with rising prevalence rates is observed in Europe, with obesity affecting between 4 and 28% of men and between 6 and 36% of women [3]. In parallel to the rising prevalence of obesity in the general population, an increasing number of overweight and obese trauma patients are treated at major trauma centers.

A number of studies have sought to describe the effect of obesity on treatment and outcome in trauma patients. Several studies describe a higher risk of mortality in blunt trauma, a higher rate of organ dysfunction and failure, as well as a prolonged ICU and hospital stay [4-7]. Ciesla and colleagues found a correlation between BMI and subsequent organ failure in their study of 716 trauma patients, irrespective of injury severity or the number of blood transfusions [6]. Bercault and colleagues reported an increased surgical ICU mortality rate especially among younger obese patients using a matched cohort study design, and attributed the excess mortality mainly to secondary complications specific to ICU treatment [4]. However, these findings are not unquestioned: neither Ciesla and colleagues or Duane and colleagues, in their series of 453 trauma patients, found differences in overall mortality in patients stratified by BMI [6,8] - only a slightly longer hospital stay was observed in obese patients, thereby raising doubts about the extent of obesity-related risks in trauma care.

The aims of our current study were threefold. Firstly, we wished to examine the distribution of normal-weight patients, obese patients, and also underweight patients in our patient population representative of a Western European trauma center and place our data in perspective with the mostly American literature. Secondly, we sought to compare admission physiologic status and treatment and in-hospital outcome parameters between underweight, normal-weight, and overweight patients. Finally, we wished to identify specific process measures that may help explain BMI-related outcome differences in trauma patients.

## Materials and methods

### Study design and setting

The study was conducted as a retrospective cohort analysis using a prospectively led single-center trauma database. The University of Zurich trauma database comprises all severe trauma patients admitted between 1996 and present. Inclusion criteria for this study are an Injury Severity Score (ISS) ≥ 16 in adult patients older than 16 years of age. Excluded from this study were patients with burn injuries and delayed referrals > 12 hours after trauma. BMI values were assessed using either information from recent hospital visits, information provided by the patient or their primary care physician, or (least preferred) by measurements taken during the patient's hospital stay. Patients with missing BMI values were excluded. A total of 1,084 patients have been enrolled into this study, which has been approved by the University of Zurich institutional review board. A waiver of individual patient consent was provided in adherence to institutional regulations, and the study was conducted according to good clinical practice guidelines.

### Definition of obesity

Patients were grouped by BMI (body mass in kilograms divided by the square of the height in meters) into five groups as defined by the World Health Organization [1]: underweight, all patients with BMI < 18.5; normal, all patients with BMI of 18.5 to 24.9; overweight, all patients with BMI of 25 to 29.9; obese, all patients with BMI of 30 to 39.9; and morbid obese, all patients with BMI ≥ 40. Because there were only 11 patients with BMI ≥ 40, we combined obese and morbidly obese patients into one group.

### Process and outcome measures analyzed

Patient demographics, injury pattern and severity, prehospital resuscitation, and diagnostic measures upon admission and their results were recorded. The patients' trauma load and initial medical condition were assessed using the Abbreviated Injury Score, ISS and New ISS, Glasgow Coma Scale, and the Acute Physiologic and Chronic Health Evaluation score. Subsequent operations as well as their duration and daily routine laboratory parameters were recorded. We further analyzed the length of time between injury, trauma room admission, transfer to the operating theater, and admission to the trauma ICU. Outcome parameters included length of hospital stay and ICU stay, length of mechanical ventilation, overall mortality, cause of death and morbidity grouped into infectious complications and multiple organ dysfunction/multiple organ failure according to the Marshall score, the Goris score, the Murray score and Sequential Organ Failure Assessment (SOFA) scores [9-11]. The Marshall and Goris scores vary in the number of organ systems evaluated and the points given to each - seven organs with 0 to 2 points each (maximum 14) in the Goris score, and six organs with 0 to 4 points each (maximum 24) in the Marshall score. The SOFA differs from the Marshall score in its definition of cardiovascular failure (for example, including amount of inotropes given). The Murray score is used to assess acute lung injury leading to acute respiratory distress syndrome [11]. Systolic blood pressure < 90 mmHg was used as a substitute definition of shock in this study to allow for quantitative comparison between groups of different BMI.

Volume therapy data are provided as crystalloid, colloid, and total volumes administered over time. Volume corrected per BMI is defined as the mean amount divided by the mean BMI in each group, thereby controlling for BMI. Arterial lactate is shown as the admission value, the percentage of patients with admission lactate above a defined threshold of 2.5 mmol/l, and the course of arterial lactate over time as a function of BMI.

### Admission day (day 0) trauma care protocols

All patients were resuscitated and diagnosed according to advanced trauma life support guidelines with routine basic imaging containing plain chest and pelvic radiography as well as focused abdominal sonography in trauma by the trauma resident. Our protocol of intravenous access provides for the use of an intraosseus cannula after the third failure to achieve intravenous access. In hemodynamically stable patients with no need for immediate operative intervention, computed tomography scanning was performed. In hemodynamically unstable patients, life-saving surgery either in the trauma room or in the adjacent operating theater was performed without prior computed tomography scanning. Our operating tables are certified for patients weighing ≤ 250 kg, thereby rarely necessitating specialized tables for bariatric surgery (for which our hospital is certified). Following initial stabilization, all patients with severe trauma were transferred to a specialized trauma ICU. Hemodynamic monitoring using an arterial radial line was used in patients with anticipated fluctuations in intravascular volume, those with pre-existing cardiac and pulmonary constraints, and in patients with traumatic brain injury. For non-invasive monitoring, an oversized cuff was used in obese patients or those with corpulent upper arms.

### Statistical analysis

Univariate comparisons between groups were performed to detect differences between the four BMI groups; analysis of variance was used to detect differences in normally distributed continuous data, and the Kruskal-Wallis-test for non-normally distributed parameters. To compare nominal data, we utilized the Pearson chi-square test or the Fisher's exact test. To correct for multiple testing among different subgroups, these tests were followed by Bonferroni correction and significance was set at *P *< 0.0083. Data are presented as the median (range) or mean ± standard deviation, as appropriate. *P *values in the tables indicate comparison of all groups demonstrating an overall effect of BMI. Error bars in the figures indicate the standard error of the mean. Independent predictors for death were determined using a stepwise logistic regression analysis. For the logistic regression only, (severe) trauma to a distinct organ was defined by Abbreviated Injury Score ≥ 3.

All statistical analyses were performed using IBM SPSS Statistics 18.0 software (SPSS Inc., Chicago, IL, USA). Graphical visualization was performed using SigmaPlot 9.0 (Systat Software Inc., Richmond, CA, USA).

## Results

### Study population

An overview of the study population is provided in Table 1. A total of 1,084 patients were included into this study, 603 (55.6%) of which are of normal weight; 2.8% of our patient collective is underweight, 33.3% are overweight and a comparably low 8.3% of patients are obese. In underweight patients (BMI < 18.5) only 33.3% were of male gender, whereas male gender dominated in all other BMI groups. Motor vehicle accidents were the most common cause of injury in all groups, followed by attempted suicide and falls in underweight patients, which occurred significantly more often in this group than in any other. ISS was lowest in underweight patients, but neither New ISS nor individual Abbreviated Injury Score scores differed significantly between the groups. The admission blood pressure and heart rate was equal in all groups. Also, neither lactate on admission as a marker of increased anaerobic metabolism nor incidence of hypothermia differed between the groups, although there was a nonsignificant trend towards rising lactate levels and less frequent hypothermia with increasing BMI (Table 1). Similarly, Acute Physiologic and Chronic Health Evaluation II scores were lowest in patients with normal BMI and showed a biphasic increase in both underweight and overweight/obese patients with highest values in obese patients, although again the differences did not attain statistical significance.

**Table 1:** Overview of the patient collective

	Body mass index				
		
	< 18.5	18.5 to 24.9	25 to 29.9	≥ 30	*P *value
Number of patients	30 (2.8%)	603 (55.6%)	361 (33.3%)	90 (8.3%)	
Age (years)	44.2 ± 20.1	39.4 ± 17.9	46.1 ± 16.6**^†^**	47.8 ± 15.4	**< 0.001**
Gender: male (%)	33.3**^‡^**	71.1	82.0**^†^**	77.8	**< 0.001**
Blunt trauma (%)	90.0	92.2	90.9	84.4*	**0.012**
Type of injury (%)					
Motor vehicle accident	40.0	50.1	49.9	48.9	0.75
Work	3.3	9.8	16.3^†^	14.4	**0.008**
Sport	3.3	8.6	7.5	4.4	0.41
Fall	20.0	11.4	10.5	12.2	**0.035**
Suicide	23.3**^‡^**	8.8	5.0**^†^**	10.0	**0.002**
Injury Severity Score	24.9 ± 8.7**^‡^**	30.9 ± 11.8	31.2 ± 12.3	29.9 ± 11.0	**0.022**
New Injury Severity Score	36.4 ± 16.3	40.4 ± 14.2	40.5 ± 15.7	40.2 ± 14.3	0.35
Admission SBP	124.0 ± 19.8	131.8 ± 26.2	130.7 ± 29.4	132.0 ± 35.6	0.06
Admission heart rate	91 ± 20.4	92.7 ± 23.1	89.7 ± 21.3	95.7 ± 25.8	0.14
Admission GCS	10.4 ± 5.4	19.7 ± 5.4	9.3 ± 5.6	9.7 ± 5.5	0.77
Admission lactate	3.1 ± 2.5	2.8 ± 2.2	3.0 ± 2.4	3.2 ± 2.4	0.4
Admission pH	7.32 ± 0.02	7.33 ± 0.01	7.31 ± 0.01**^†^**	7.29 ± 0.02*	**0.001**
Admission temperature (°C)	35.1 ± 1.4	35.5 ± 1.8	35.6 ± 1.4	35.9 ± 1.1	0.19
Admission APACHE II	14.1 ± 7.4	13.4 ± 8.1	14.9 ± 8.7**^†^**	15.3 ± 8.6	**0.03**
Hospital stay (days)	23.3 ± 14.0	22.0 ± 18.0	25.2 ± 31.6	24.5 ± 22.6	0.78
ICU stay (days)	9.8 ± 8.3	10.0 ± 11.1	11.2 ± 12.7	12.4 ± 14.5	0.49
Mechanical ventilation (days)	6.2 ± 7.4	5.9 ± 8.0	7.1 ± 10.2	7.7 ± 10.4	0.44

P-values < 0.05 in bold. APACHE, Acute Physiology and Chronic Health Evaluation Score; GCS, Glasgow coma scale; SBP, systolic blood pressure. *P *values indicate comparisons between all BMI groups. *Significant differences between BMI of 18.5 to 24.9 and BMI ≥ 30. **^†^**Significant differences between BMI of 18.5 to 24.9 and BMI of 25 to 29.9. **^‡^**Significant differences between BMI of 18.5 to 24.9 and BMI < 18.0.

The time between injury and admission (average 2.2 ± 2.7 hours in the entire collective), the time from admission to initial operation (2.1 ± 2.5 hours), the time to ICU admission (4.6 ± 2.6 hours), or the length of the initial operation (116 ± 107 minutes) did not differ significantly in any BMI group (all *P *> 0.3).

### Mortality as a function of body mass

**Figure 1:**
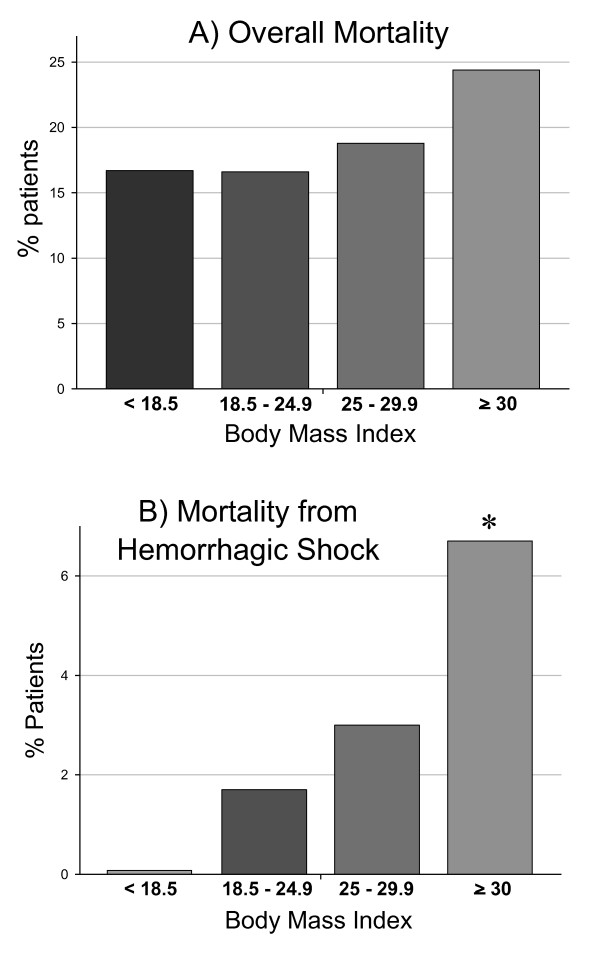
**Mortality as a function of body mass index**. **(A) **Overall mortality and **(B) **mortality from hemorrhagic shock. *Significant differences between patients with body mass index of 18.5 to 24.9 compared with patients with BMI > 30.

Unadjusted mortality rates as a function of BMI are shown in Figure 1. Normal-weight patients have a mortality of 16.6%, which increases to 18.8% in patients with BMI ≥ 25 and to 24.4% in patients with BMI ≥ 30, even though this increase is not statistically significant (Figure 1A). However, obese patients had a higher mortality on day 0 compared with normal-weight subjects (8.9% vs. 2.8% in the normal-weight group, *P *= 0.023). Among nonsurvivors, we found that six obese patients (6.7%) died from hemorrhagic shock, as opposed to only 3% of overweight patients and only 1.7% of normal-weight patients (*P *= 0.024; Figure 1B). These six patients (three of which committed suicide) all suffered from pelvic ring injuries and/or severe thoracic and abdominal injuries (average ISS 32). All except one patient underwent immediate surgical exploration to control bleeding (in three patients prior to computed tomography scanning), but all six died within a maximum 4 hours after admission (Additional File 1).

Logistic regression showed that BMI ≥ 30 was an independent predictor of mortality in the entire cohort, next to elevated lactate, traumatic brain injury, and New ISS > 50 (Table 2).

**Table 2:** Independent predictors of mortality

All patients	Odds ratio	95% CI	Patients with BMI ≥ 30	Odds ratio	95% CI
BMI ≥ 30	2.52	1.3 to 4.9	New ISS > 50	4.75	1.2 to 19.0
Traumatic brain injury^a^	3.09	1.7 to 5.5			
Admission lactate > 2.5 mmol/l	2.61	1.6 to 4.4			
New ISS > 50	2.95	1.7 to 5.1			

BMI, body mass index; CI, confidence interval; ISS, Injury Severity Score. ^a^Defined as head Abbreviated Injury Score ≥ 3.

### Prevalence of shock on admission and volume therapy

**Figure 2:**
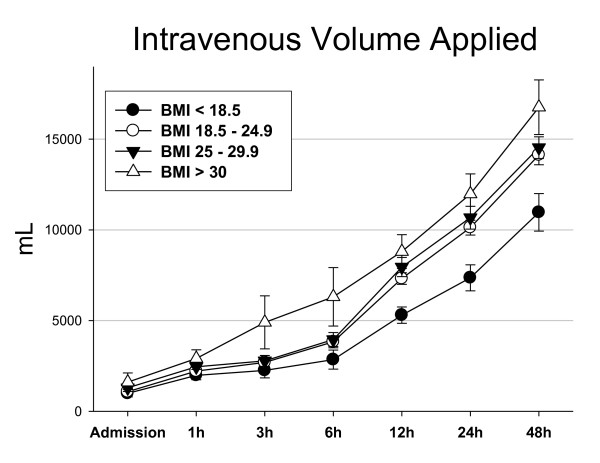
**Total amount of intravenous volume applied**. Error bars indicate the standard error of the mean.

**Figure 3:**
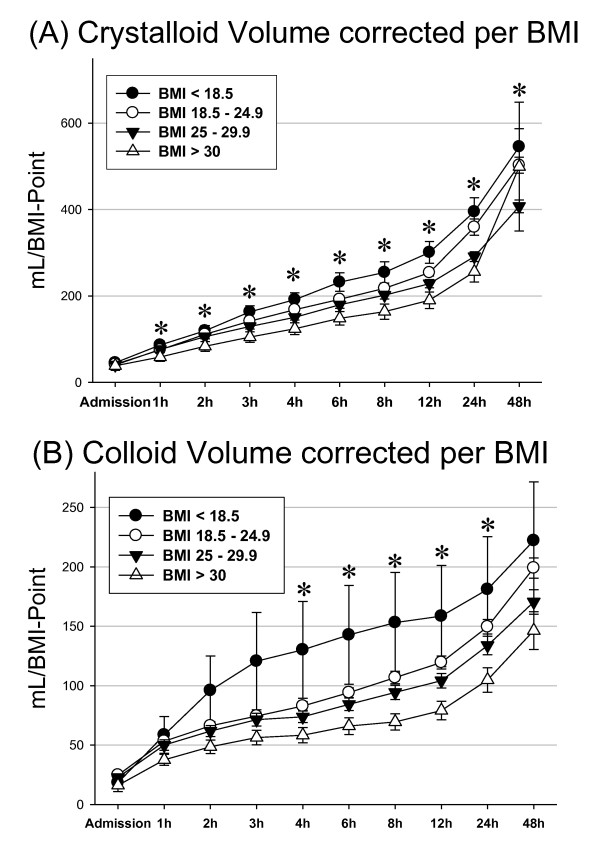
**Crystalloid and colloid volume per body mass index point**. Amount of **(A) **crystalloid volume and **(B) **colloid volume corrected for body mass index (BMI) point (total amount/number of BMI points) per BMI group. Error bars indicate the standard error of the mean. *Significant differences between patients with BMI of 18.5 to 24.9 compared with patients with BMI > 30.

The percentage of patients admitted with systolic blood pressure < 90 mmHg was lowest in underweight patients (6.7%) and rose to 14.9% in obese patients (*P *= 0.55). When looking at gross intravenous volumes given during the initial resuscitation, we found a parallel increase over time with highest volumes applied in obese patients and lowest volumes in underweight patients (Figure 2). When correcting for BMI (Figure 3A, Figure 3B), however, we found that obese patients receive significantly lower volumes of intravenous fluids than their normal-weight or even underweight counterparts; this finding is true for both crystalloid (Figure 3A) and colloid (Figure 3B) intravenous fluids, and is most prominent between 4 and 24 hours following trauma. For example, obese patients were given an average 69.5 ml/BMI point of colloid fluids at 8 hours after injury compared with 153.1 ml/BMI point in underweight patients, or 55% less. The differences in milliliters of crystalloid fluids/BMI point remain significant up to 48 hours after admission (Figure 3A), the latest time point analyzed in this study.

**Figure 4:**
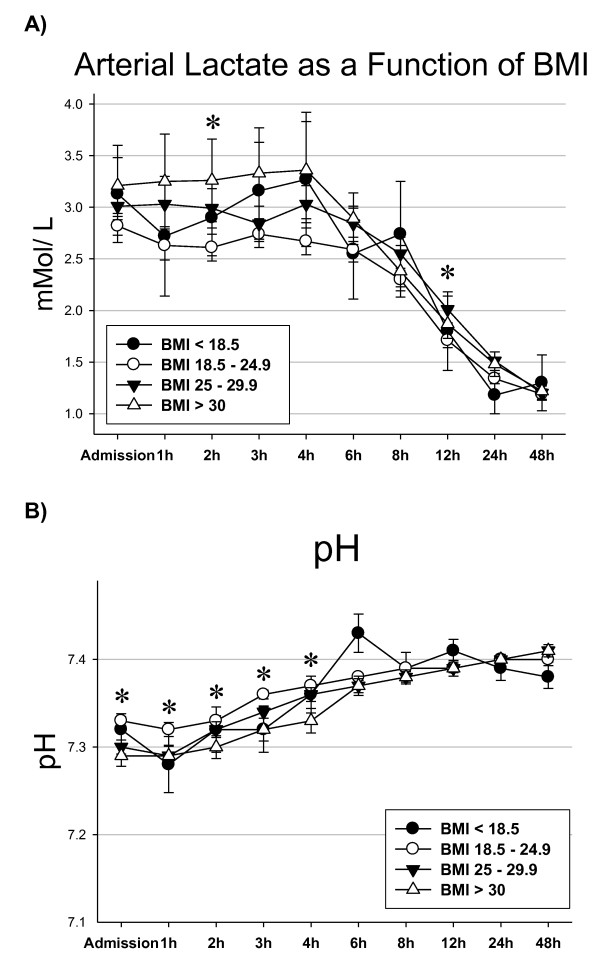
**Parameters of resuscitation**. **(A) **Arterial lactate and **(B) **pH. Error bars indicate the standard error of the mean. *Significant differences between patients with body mass index (BMI) of 18.5 to 24.9 compared with patients with BMI > 30.

The courses of arterial lactate and pH are shown in Figure 4A and 4B, respectively, showing optimum pH levels in normal-weight patients and a trend towards higher lactate levels in patients with BMI ≥ 30, although this is only significant at one time point. The percentage of patients with arterial lactate levels ≥ 2.5 mmol/l on admission is lowest (43.1%) in patients with normal BMI and is highest (51.8%) in patients with BMI ≥ 30 (*P *= 0.52).

### Organ failure scores as a function of body mass

Table 3 provides an overview of different commonly used scores of organ dysfunction and failure: the Marshall score, the Murray score and the SOFA score all show lowest values in either underweight or normal-weight patients, with a subsequent significant increase in parallel to rising BMI levels. This observation applies to both admission and maximum values in the course of the hospital stay. In parallel, peak systemic inflammatory response syndrome levels are lowest in patients with normal BMI, and higher in both underweight and overweight groups.

**Table 3:** Overview of multiple organ failure scores

	Body mass index				
		
Organ failure score	< 18.5	18.5 to 24.9	25 to 29.9	≥ 30	*P *value^a^
Marshall score					
Admission	3.9 ± 2.7	4.2 ± 3.0	4.7 ± 3.3	5.2 ± 3.4*	**0.013**
Maximum	4.9 ± 3.6	5.0 ± 3.5	6.0 ± 4.1	6.5 ± 3.7*	**< 0.001**
Goris score					
Admission	4.0 ± 2.1	4.2 ± 2.3	4.3 ± 2.4	4.8 ± 2.7	0.37
Maximum	4.6 ± 2.5	4.4 ± 2.5	4.7 ± 2.5	5.1 ± 2.7	0.06
SOFA score					
Admission	5.5 ± 3.6	5.8 ± 3.8	6.1 ± 4.0	6.8 ± 4.2	0.24
Maximum	6.3 ± 4.2	6.8 ± 4.4	7.5 ± 4.7	7.9 ± 4.4*	**0.032**
Murray score					
Admission	0.9 ± 0.9	0.9 ± 1.1	1.1 ± 1.1	1.1 ± 1.0	0.08
Maximum	1.3 ± 0.9	1.5 ± 1.4	1.7 ± 1.3	1.1 ± 1.9*	**0.001**
SIRS score					
Admission	2.3 ± 1.1	2.4 ± 1.1	2.3 ± 1.1	2.5 ± 1.1	0.24
Maximum	2.7 ± 1.3	2.4 ± 1.4	2.6 ± 1.3	2.7 ± 1.3	0.07

P-values < 0.05 in bold. SIRS, systemic inflammatory response syndrome; SOFA, Sequential Organ Failure Assessment. ^a^*P *value indicates overall comparisons between all BMI groups. BMI of 18.5 to 24.9 serves as the control group. *Significant differences between BMI of 18.5 to 24.9 and BMI ≥ 30.

## Discussion

Trauma in obese patients has become a regular occurrence in western trauma centers and the topic has recently been given broad attention in the North American literature. By nature, the percentage of obese patients varies greatly between studies (5 to 31%), with a higher incidence in more recent reports [5-7,12-18]. However, fewer data exist on the incidence of obesity in European trauma centers. The first aim of our study was therefore to survey the incidence of obesity in our trauma population, which appears comparably low at a mean of 8.3% from the years 1996 to 2009. In fact, only 11 patients (1%) of our population fall into the category of morbid obesity. At the same time, our percentage of overweight patients (35% with BMI of 25 to 29.9) is similar to that in the existing literature [5-7,12,15,16,18].

In essence, our study shows that obesity is an independent predictor for overall mortality after trauma (Table 2). Unadjusted mortality rates are highest in obese patients and lowest in normal-weight patients. Underweight patients (BMI < 18.5) had nonsignificantly higher mortality, which has been described earlier [19,20]. Interestingly, the rate of suicide was almost threefold higher in underweight patients when compared with normal-weight patients. The association between BMI and trauma-related mortality is discussed controversially in the literature; most authors report higher mortality rates in obese trauma patients, such as Neville and colleagues who surveyed 242 blunt trauma patients and observed a mortality rate of 32% in obese patients compared with 16% in normal-weight patients [14]. Similarly, Choban and colleagues reported a high (42.1%) mortality rate among extremely overweight (BMI ≥ 40) patients compared with only 5% among those with BMI < 27 [21]. Bercault and colleagues showed an increased mortality in obese patients in a large study of 1,927 patients (32% vs. 17%) [4]. A recent analysis of 5,766 patients from the German Trauma Registry also showed a significant correlation between BMI and mortality in both overweight and underweight patients [19].

However, several well-designed trials have been unable to confirm higher mortality rates in obese patients [6,8]. In a prospective study on 716 trauma patients, Ciesla and colleagues found an almost twofold higher incidence of early multiple organ failure and a prolonged hospital stay in obese patients, but no difference in overall mortality [6]. Similarly, Duane and colleagues in their retrospective analysis of 338 trauma victims did not record a higher mortality in obese patients [8]. A recent meta-analysis on the effects of obesity on survival in critically ill surgical patients by Akinnusi and colleagues has shown equal mortality in obese and nonobese patients, which the authors attributed to several factors. First, they argued that the abundance of adipose tissue may diminish the consequences of the severe catabolic state in prolonged systemic illness [22]. Alternatively, obesity may already contribute to greater alertness among ICU staff and expedite the correction of physiologic derangements such as stress hyperglycemia. Akinnusi and colleagues' study also found that obese patients required a prolonged length of ICU stay and mechanical ventilation. The authors attribute these findings to a diminished respiratory reserve in obese patients and a subsequent risk of respiratory failure following even mild pulmonary or systemic insults [22]. In fact, only three of the examined 13 studies did not show a prolonged ICU length of stay in obese patients. Our data are in accordance with these findings in that we recorded (nonsignificantly) longer lengths of stay in obese and overweight patients, which may be explained by a higher rate of comorbidities (Acute Physiologic and Chronic Health Evaluation II scores) and more frequent complications such as superficial wound infections.

Similarly, the occurrence of multiple organ dysfunction and multiple organ failure appears to be associated with obesity (Table 3): the Marshall score, the Murray score, or the SOFA score show higher values with increasing BMI either at admission or as a peak value, indicating a moderate yet significant level of organ dysfunction. These findings are comparable with a recent study of 1,543 trauma patients by Newell and colleagues in which the authors found an odds ratio of 13.5 for the development of renal failure in morbidly obese patients [23]. Hence, the higher incidence of renal failure may be due to an unrecognized need for higher fluid requirements in obese trauma victims.

Another finding of our study is that obesity is associated with increased mortality from hemorrhagic shock, despite this being a rare occurrence. In fact, no underweight patients and only 10 patients with a normal BMI died from this cause. Our data suggest that, once controlled for BMI, the initial volume therapy may be inadequate: arterial pH values showed optimal courses only in patients with normal BMI. In the same way, we observed a higher rate of hypotension on admission and a greater percentage of obese patients with lactate levels above 2.5 mmol/l, although neither difference reached statistical significance.

Initial volume therapy is guided by parameters such as hemodynamic monitoring, arterial lactate, diuresis, and visible and anticipated blood loss. Monitoring cardiac output/index using Swan-Ganz or peripherally inserted central catheters may help to assess individual hemodynamics and distinguish hypovolemic from distributive shock, but their use in trauma care is debatable [23]. Our data indicate that metabolic derangements in obese patients are not reflected in significantly lower systolic or mean arterial blood pressures, thereby facilitating inadvertent under-resuscitation. Difficulty in intravenous access with a need for intraosseous cannulation may also further impair resuscitation, but this was not analyzed in our study.

In their study on 625 trauma patients, Belzberg and colleagues showed reduced cardiac index and impaired tissue oxygenation with early organ failure in obese nonsurvivors when compared with obese survivors [24]. They emphasized the need for continuous monitoring in obese patients using the cardiac index, central venous pressure, and peripheral tissue oxygenation as markers of adequate resuscitation. Correspondingly, Winfield and colleagues concluded in their study of 877 trauma patients that morbidly obese patients are inadequately resuscitated during the first 48 hours [18]. These patients remained in metabolic acidosis longer, resolved their base deficit more slowly, and developed multiple organ failure at a higher rate than normal-weight patients. In this study population, nearly 90% of obese patients with ongoing metabolic acidosis developed multiple organ failure. The authors argue that pH may provide a more reliable marker due to the large variations inherent in central venous pressure and the influence of inorganic acids on metabolic acidosis, rendering lactate a less suitable parameter during the early resuscitation period.

## Conclusions

Our study indicates that trauma of the obese is less frequent at a European trauma center when compared with the averages outlined in the American literature. Obese patients had a higher mortality due to persistent hemorrhagic shock on admission, despite comparable injury severity. Inadequate resuscitation resulting from an underestimation of individual volume requirements may add to this risk. Our data further indicate that restrictive fluid management may sustain unresolved metabolic acidosis in obese patients and may be associated with a higher rate of MOF. Future studies are needed to identify as yet unknown factors that promote metabolic acidosis in obese trauma patients and to establish reliable markers for the continuous monitoring of under-resuscitated individuals.

## Key messages

• Highest mortality on day 0 is observed in obese patients.

• Uncontrolled hemorrhage is the leading cause of death in obese patients.

• Protracted hypoperfusion with metabolic acidosis should prompt expeditious resuscitation, preferably with invasive hemodynamic monitoring.

• Restrictive volume therapy may be potentially hazardous in obese trauma patients.

## Abbreviations

BMI: body mass index; ISS: injury severity score; SOFA: Sequential Organ Failure Assessment.

## Competing interests

The authors declare that they have no competing interests.

## Authors' contributions

JN carried out the data acquisition, participated in subsequent data analyses, and provided a first manuscript draft. ATB helped in data acquisition and conducted initial statistical analyses, provided the figures and tables, and helped draft the manuscript. BS participated in the study design/supervised all statistical analyses and performed additional analyses where necessary, and helped author the statistics section. VN assisted in designing the study as well as analyzing and interpreting the data, and critically revised the final manuscript. OT helped in data interpretation and critically revised the manuscript. CH assisted in data interpretation and analysis as an expert in anesthesiology and intensive care medicine, and helped to write the manuscript. MT conceived the study, supervised data acquisition and analysis, and wrote the final manuscript. All authors read and approved the final version of the manuscript.

## Supplementary Material

Additional file 1 **Table presenting an overview of the sustained injuries, status on admission, and the course of treatment of the six obese patients who died as a result of hemorrhagic shock on the day of admission**. Click here for file
