# Supplementary material for: Obese trauma patients are at increased risk of early hypovolemic shock: a retrospective cohort analysis of 1,084 severely injured patients
Source: Crit Care. 2012 May 8;16(3):R77. doi: 10.1186/cc11334 (PMC3580619; doi:10.1186/cc11334)
Supplement: Additional file 1 — Table presenting an overview of the sustained injuries, status on admission, and the course of treatment of the six obese patients who died as a result of hemorrhagic shock on the day of admission. [file cc11334-S1.DOCX]

| **Patient** | **ISS** | **Leading Injuries** | **Admission**  **Vital Signs** | **Surgical Therapy** | **Volume Therapy** | **Time to Death** |
| --- | --- | --- | --- | --- | --- | --- |
| **Patient 1**  MVA  BMI 37 | ISS: 57  NISS: 66 | Bilateral hemothorax  Splenic rupture with portal vein injury  Serial fractures of lower extremities | Syst. BP: 70  HR: 135  Hb: 4.6  Lac: 9.1 | Chest tubes  Chest packing  Splenectomy & portal vein repair  External fixation | Total fluids: 7500  Units of blood: 39  FFP: 37  NovoSeven: 7.2mg | 4h |
| **Patient 2**  Work accident  BMI: 33.4 | ISS: 35  NISS: 38 | Pelvic ring injury with massive retroperitoneal hematoma and abdominal compartment syndrome | Syst. BP: 150  HR: n/a  Hb: 7.2  Lac: 5.4 | Exploratory laparotomy  Pelvic packing | Total fluids: 8800  Units of blood: 8  FFP: 6 | <1h |
| **Patient 3**  Suicide  BMI: 31.3 | ISS: 18  NISS: 27 | Hemopneumothorax  Pelvic ring injury | Syst. BP: 150  HR: 120  Hb: 7.6  Lac: 8.3 | Thoracotomy  Open cardiac massage | Total fluids: 3800  Units of blood: 9  FFP: 0 | 1h |
| **Patient 4**  MVA  BMI: 31.2 | ISS: 38  NISS: 57 | Hemopneumothorax  Humerus fracture | Syst. BP: 160  HR: n/a  Hb: 8.2  Lac: 2.7 | Chest packing  Open cardiac massage  External fixation | Total fluids: 20100  Units of blood: 30  FFP: 28 | 3h |
| **Patient 5**  Suicide  BMI: 31 | ISS: 25  NISS: 50 | Hemopneumothorax  Liver rupture  Colonic laceration | Syst. BP: 85  HR: 40  Hb: 13.4  Lac: 4.5 | Chest packing  Open cardiac massage  Segmental colon resection &  colostomy  Abdominal packing | Total fluids: 8000  Units of blood: 12  FFP: 4 | 3h |
| **Patient 6**  Suicide  BMI: 31 | ISS: 19  NISS: 34 | Hemopneumothorax  Spinal injury  Pelvic ring injury | Syst. BP: 120  HR: 103  Hb: 11.0  Lac: 1.8 | Chest tubes, then withdrawal of medical support | Total fluids: 5500  Units of blood: 0  FFP: 0 | 3h |

**Additional File 1: Obese Patients Deceased From Hemorrhagic Shock**

MVA, motor vehicle accident. BMI, body mass index. (N)ISS; (new) injury severity score. BP, blood pressure. HR, heart rate. Hb, hemoglobin. Lac, arterial lactate. FFP, fresh frozen plasma.
